# Glucose transporter 1 critically controls microglial activation through facilitating glycolysis

**DOI:** 10.1186/s13024-019-0305-9

**Published:** 2019-01-11

**Authors:** Luxi Wang, Sofia Pavlou, Xuan Du, Mohajeet Bhuckory, Heping Xu, Mei Chen

**Affiliations:** The Wellcome-Wolfson Institute of Experimental Medicine, School of Medicine, Dentistry and Biomedical Sciences, Queen’s University Belfast, 97 Lisburn Road, Belfast, BT9 7BL UK

**Keywords:** Neuroinflammation, Microglia, Glucose metabolism, Retinal degeneration

## Abstract

**Background:**

Uncontrolled microglial activation contributes to the pathogenesis of various neurodegenerative diseases. Previous studies have shown that proinflammatory microglia are powered by glycolysis, which relays on high levels of glucose uptake. This study aimed to understand how glucose uptake is facilitated in active microglia and whether microglial activation can be controlled by restricting glucose uptake.

**Methods:**

Primary murine brain microglia, BV2 cells and the newly established microglial cell line B6M7 were treated with LPS (100 ng/ml) + IFNγ (100 ng/ml) or IL-4 (20 ng/ml) for 24 h. The expression of glucose transporters (GLUTs) was examined by PCR and Western blot. Glucose uptake by microglia was inhibited using the GLUT1-specific inhibitor STF31. The metabolic profiles were tested using the Glycolysis Stress Test and Mito Stress Test Kits using the Seahorse XFe96 Analyser. Inflammatory gene expression was examined by real-time RT-PCR and protein secretion by cytokine beads array. The effect of STF31 on microglial activation and neurodegeneraion was further tested in a mouse model of light-induced retinal degeneration.

**Results:**

The mRNA and protein of GLUT1, 3, 4, 5, 6, 8, 9, 10, 12, and 13 were detected in microglia. The expression level of GLUT1 was the highest among all GLUTs detected. LPS + IFNγ treatment further increased GLUT1 expression. STF31 dose-dependently reduced glucose uptake and suppressed Extracellular Acidification Rate (ECAR) in naïve, M(LPS + IFNγ) and M(IL-4) microglia. The treatment also prevented the upregulation of inflammatory cytokines including TNFα, IL-1β, IL-6, and CCL2 in M(LPS + IFNγ) microglia. Interestingly, the Oxygen Consumption Rates (OCR) was increased in M(LPS + IFNγ) microglia but reduced in M(IL-4) microglia by STF31 treatment. Intraperitoneal injection of STF31 reduced light-induced microglial activation and retinal degeneration.

**Conclusion:**

Glucose uptake in microglia is facilitated predominately by GLUT1, particularly under inflammatory conditions. Targeting GLUT1 could be an effective approach to control neuroinflammation.

**Electronic supplementary material:**

The online version of this article (10.1186/s13024-019-0305-9) contains supplementary material, which is available to authorized users.

## Background

Microglia are resident macrophages of the central nervous system (CNS) including the neuronal retina [1, 2]. As innate immune cells, microglia can initiate immune protection under pathophysiological conditions to remove metabolic wastes, cellular debris and pathogens [2–4]. Under physiological conditions, the debris in the CNS microenvironment is largely derived from active neurons, including apoptotic bodies and synaptic debris [5]. Microglia are actively involved in synaptic pruning and synapse maturation [6], therefore play an important role in the regulation of synaptic plasticity [7, 8]. During infection, microglia can phagocytize pathogens and remove dead cells to maintain homeostasis [9]. Uncontrolled microglial activation has been implicated in neurodegenerative diseases such as Huntington disease, multiple sclerosis, Alzheimer’s disease [10], and retinal degeneration [2].

A dynamic cell body and flexibility to rapid change shape are essential for microglia to carry out their protective roles. The protrusions of “resting” microglia are continuously moving around, scanning the CNS [11, 12]. After activation, microglia rapidly extend processes towards the damaged site and retract processes facing the other direction [13]. The processes are employed to phagocytize cellular debris and damaged neurons [14]. Once engaged with pathogens, they become fully activated, obtain an “ameboid” shape and can move around rapidly. In addition, microglia release various cytokines and chemokines, and can proliferate in situ in response to increased demand in immune protection [15]. Morphogenesis, phagocytosis, and migration require dynamic reorganization of the actin cytoskeleton, for which vast amount of energy in the form of adenosine triphosphate (ATP) are necessary [16]; whereas, proliferation and the production of inflammatory mediators need essential “building materials” such as amino acids, nucleotides, and fatty acids. How microglia meet their metabolic demands is not fully understood.

Glucose, amino acids and fatty acids are the three main energy substrates. Each cell type has its own unique metabolic profile and is fueled by specific energy substrates under physiological conditions, and changes in cellular activity can induce a switch of this metabolic profile. A previous study using RNA-sequencing technique has shown that microglia express transporters for all these substrates, indicating a flexible use of available energy resources [17]. However, which metabolic pathway dominates the energy resource in microglia under pathophysiological conditions remains unknown.

Recent studies have shown that LPS-stimulated inflammatory microglia switch from mitochondrial oxidative phosphorylation (OXPHOX) in resting conditions to anaerobic glycolysis [18], a phenomenon that is similar to active macrophages [19]. The mammalian brain utilizes glucose as its main source of energy [20], and the majority of CNS glucose is consumed by neurons through the glucose transporter 3 (GLUT3) [20]. During CNS inflammation, the high level of glycolysis in active microglia not only supports the production of toxic inflammatory mediators, but also consumes vast amount of glucose that is desperately needed by neurons. Further understanding the metabolic pathways of active microglia will be important to better control neuroinflammation and improve the management of neurodegeneration.

In this study, we show that microglia express high levels of glucose transporter 1 (GLUT1), which critically controls glucose uptake under pathophysiological conditions. Blocking GLUT1 suppressed microglial activation and reduced neurodegeneration in a mouse model of light-induced retinal degeneration.

## Materials and methods

### Animals

Four-week old wild-type C57BL/6 J mice were used for primary microglial culture. Twelve-week old C57BL/6J and *CX3CR1*^*gfp/+*^ mice (C57BL/6 J background) were used in the light-induced retinal degeneration study. All mice were maintained in the Biological Services Unit of Queen’s University Belfast (QUB) with 12-h light-dark cycle. All procedures were conducted under the regulation of the UK Home Office Animals (Scientific Procedures) Act 1986, and were in compliance with the Association for Research in Vision and Ophthalmology Statement for the Use of Animals in Ophthalmology and Vision Research. The procedures were approved by the Animal Welfare & Ethical Review Body of QUB.

### Microglial culture

BV2 cells were cultured in DMEM (Gibco, BRL, Paisley, UK) supplemented with 10% fetal calf serum (FCS) and 100 μg/ml primocin (Invivogen, San Diego, California, USA). Primary murine microglia were isolated and cultured from C57BL/6 J mice using a previously reported protocol with slight modifications [21]. Briefly, brain tissues were dissected from 4-week old mice and digested with 0.05% trypsin at 37 °C for 10 min. The single cell suspension was cultured in DMEM with 10% FCS and 20% L929 conditioned media [22]. After 5 days, floating cells were removed, and attached cells (microglia) were cultured for a further 5 days. The cells were passaged once reached confluency, and cells from passages 2–5 were used in the study. Immunocytochemistry showed that > 95% of cells were CD11b^+^F4/80^+^. During primary microglia culture, one cell line was spontaneously immortalized and subsequently cloned. One of the clones, B6M7 was also used in this study.

### Cell stimulation

BV2, B6M7 and primary microglia were treated with LPS (100 ng/ml, *E. coli*, serotype 055: B5) + IFNγ (100 ng/ml) or IL-4 (20 ng/ml) (all from Sigma-Aldrich, Dorset, UK) for 24 h. The cells were termed as M(LPS + IFNγ) and M(IL-4) respectively. The supernatants were collected for cytokine/chemokine measurements (see below). Cells were collected for gene and protein expression studies.

### Quantitative real-time reverse transcription PCR

Total RNA was extracted from BV2, B6M7 or primary microglia using TRI Reagent (Sigma-Aldrich). cDNA synthesis was performed with a random primer using the SuperScript™ II Reverse Transcriptase kit (Invitrogen) in a reaction of 1 μg of total RNA per reaction. Murine mRNA expression levels were quantified by qRT-PCR using SYBR Green I Master and the LightCycler 480 system (Roche Diagnostics GmbH, Mannheim, Germany) with specific primers (Table 1). GLUT1–13 primers were purchased from Primer Design (Southampton, UK). Other primers were self-designed using the Primer-Blast (https://www.ncbi.nlm.nih.gov/tools/primer-blast/) and purchased from Integrated DNA Technologies, Inc. (Leuven, Belgium).

**Table 1:** Primers used for qRT-PCR

Targets	Annealing temperature (°C)	Sequences (5′ - 3′) F: forward R: reverse
β-actin	58	F CCTTCCTTCTTGGGTATG
		R TGTAAAACGCAGCTCAGTAA
GLUT1	60	F CCCCGTCCTGCTGCTATTG
		R GCACCGTGAAGATGATGAAGAC
GLUT3	60	F TGTCCAGGAACCGATCTATGC
		R CCACCAGGAACAGAGAAACTAC
GLUT4	60	F CCAGTATGTTGCGGATGCTAT
		R TTTTAGGAAGGTGAAGATGAAGAAG
GLUT5	60	F ACCTCAGCGCAGGCGTGAAA
		R AGCAGGCTATGAGGCAGGTGGA
GLUT6	60	F CGTCCAGTTTGTGCCAAGG
		R CAGCAGGGGTATCAGGGTAAG
GLUT8	60	F GCTCACCGCTGCCTTCTG
		R CGAAATGGGCTGTGACTTGTT
GLUT9	60	F ACTATGTGGACTCAATGCGATCT
		R TCAATGACCAAGCCAGAGAAGA
GLUT10	60	F CTCTGTGGCGTTCATCAAGTG
		R ACGAGGAAGGCGGAGACT
GLUT12	60	F TGGCAGCAAAACCTTCCTCT
		R AGGGACTGGTTAAGAAGACTATGG
GLUT13	60	F CTGGAATAAACTGGATTTTCAACG
		R TCAGGGAGGCAGCCATAGA
Arginase 1	61	F TTATCGGAGCGCCTTTCTCAA
		R TGGTCTCTCACGTCATACTCTGT
CCL2	58	F AGGTCCCTGTCATGCTTCTG
		R TCTGGACCCATTCCTTCTT
IL-1β	61	F TCCTTGTGCAAGTGTCTGAAGC
		R ATGAGTGATACTGCCTGCCTGA
iNOS	60	F GGCAAACCCAAGGTCTACGTT
		R TCGCTCAAGTTCAGCTTGGT
TNF-α	58	F GCCTCTTCTCATTCCTGCTT
		R CTCCTCCACTTGGTGGTTTG
IL-12P40	60	F GACATCATCAAACCAGACCCGCC
		R GCCTTTGCATTGGACTTCGGT

### Cytokine and chemokine measurement

Cytokines and chemokines including MCP-1/CCL2, IL-6, TNF-α and CCL5/RANTES in supernatants were measured by Cytometric Bead Array (CBA) assay (BD Biosciences, Oxford, UK) according to the manufacturer’s instructions. Briefly, 25 μl sample and 25 μl capture beads were mixed and incubated for 1 h followed by the addition of 25 μl PE-conjugated detection reagent for a further hour. Samples were washed and were acquired using FACS Canto II (BD Biosciences). Data were analyzed using FlowJo software. The fold change in cytokine levels relative to control groups was calculated.

### Produciton of Reactive Oxygen Species (ROS)

Intracellular ROS was measured using CellROX Green kit; mitochondrial ROS was measured using MitoSOX (MitoSOX Red), and nitrite oxide (NO) concentration in the supernatants was measured using the Griess Reagent Kit according to the manufacturers’ instructions. All reagents were from Thermo Fisher Scientific (Winsford, UK). Fluorescent signaling for CellROX and MitoSOX and absorbance for NO was immediately measured using FLUOstar Omega plate reader (BMG Labtech Ltd., Bucks, UK).

### Glucose uptake

Cells were cultured in a 96-well plate until 80% confluence. Prior to the assay, cells were washed with PBS twice and incubated in serum-free DMEM overnight. Glucose uptake by different microglial cells was measured using the Glucose Uptake Assay Kit (ab136955, Abcam, Cambridge, UK) following manufacturer’s instructions. Glucose levels were was quantified using the FLUOstar Omega plate reader (BMG Labtech).

### Cell bioenergy tests

BV2 and B6M7 cells were seeded in XFe 96-well microplates (6000 cells/well) (Agilent Technologies, Sana Clara, USA) with/without 5 μM STF31 (C_23_H_25_N_3_O_3_S, Millipore, Watford, UK) for 24 h, followed by LPS + IFNγ (both 100 ng/ml) or IL-4 (20 ng/ml) stimulation for a further 24 h. Cells were washed and incubated in base medium (Agilent Technologies) at 37 °C for 1 h. Extracellular Acidification Rate (ECAR) and Oxygen Consumption Rate (OCR) were measured in real-time with Glycolysis Stress Test Kit and Mito Stress Test Kit respectively using the Seahorse XFe96 Analyser (Agilent Technologies) following manufacturer’s instructions. Data were normalized by cell numbers that was measured by the YO-PRO®-1 Assay (Thermo Fisher Scientific).

### GLUT1 inhibition by STF31 in vitro

Microglial cells were seeded into 96-well plates and treated with different concentrations of STF31 (Millipore) ranging from 0.01 μM to 50 μM for 24 h to 48 h. Cells were further processed for glucose uptake assay (see above) or cell viability using AlamarBlue® Assay (Thermo Fisher Scientific) following the manufacturer’s instructions.

### Phagocytosis assay

B6M7 microglial cells in 96-well plates (10,000/well) were treated with different concentrations of STF31for 24 h. The pHrodo® Green *S. aureus* BioParticles® kit (Thermo Fisher Scientific) was used to determine phagocytosis of microglia following the manufacture’s instructions. The fluorescence intensity was quantified using a plate reader (FLUOstar Omega microplate reader) at 485 nm (Excitation)/530 nm(Emission).

### TUNEL staining

Cells and tissues were fixed in 2% paraformaldehyde (PFA, Agar Scientific Ltd., Cambridge, UK) for 10 min or 2 h, respectively. Eyes were embedded in optimal cutting temperature compound (OCT, Thermo Fisher Scientific) and cryosectioned (Leica CM1950 cryostat, UK). 14 μm thick cryosections were treated with 0.1% Triton X-100 (Millipore) for 5 min at room temperature followed by 3 washes with PBS. Samples were incubated with TUNEL-MIX, followed by 5% TUNEL-Enzyme in TUNEL Label (Roche). DNase I (50 U/μl, Sigma-Aldrich) treated samples were used as positive control. Negative controls were incubated with TUNEL label only. The samples were mounted using Vectashield medium with DAPI (Vector Laboratories Ltd., Peterborough, UK).

### Light-induced retinal degeneration and STF31 administration in mice

C57BL/6 J (12-week old) mice were treated with 10 mg/kg STF31 twice daily for 2 days, followed by once daily for another 3 days. The vehicle dimethyl sulfoxide (DMSO) treated mice were used as controls. Body weight and electroretinography were measured on day 6, and eyes collected for immunohistochemistry. Four mice were used in each group.

The *CX3CR1*^*gfp/+*^ mice were treated as above with the first injection received 1 day before light exposure. Mice were dark-adapted for 16 h and pupils were dilated with 1% phenylephrine and 2.5% tropicamide (Chauvin, Essex, UK) under dim light. Mice were anesthetized with ketamine (Vetoquinol UK Ltd., Buckingham, UK) and Rompun (Bayer HealthCare, Kiel, Germany) and exposed to a focal white light (50,000 lx) delivered by an otoscope (1218AA, Karl Storz, Tuttlingen, Germany) for 10 min. At the end of the STF31 treatment, clinical and immunohistochemical investigations were performed. Five mice were used in each group.

### Spectral domain optical coherence tomography

Mice were anesthetized and pupils dilated as described above. Viscotears Liquid Gel (Novartis Pharmaceuticals Ltd., Surrey, UK) was used to moisture the cornea. OCT images (30° field of view) were collected using Spectral Domain Optical Coherence Tomography (SD-OCT, Heidelberg Engineering Ltd., Hertfordshire, UK). Neuroretinal thickness was measured in the area approximately 1000 μm away from the edge of the optical disc.

### Electroretinography

Scotopic electroretinography (ERG) was conducted in 12-week old WT C57BL/6 J mice before and after STF31 treatment using Espion Visual Electrophysiology system (Diagnosys LLC, Cambridge, UK) as described previously [23]. Briefly, mice were anesthetized and pupils dilated as above. The mouse was placed on the heat-pad (38 °C). ERG was recorded using mouse corneal ERG electrodes in response to single white light flash, delivered by a standard Ganzfeld Stimulator (Diagnosys LLC). ERG signals were bandpass filtered between 0.3–500 Hz (without notch filtering), amplified 500-fold and digitized at 2 kHz using the CMGS-1 electrophysiology system (Diagnosys LLC).

### Retinal flat mount staining

Mouse eyes were fixed in 2% PFA at room temperature for 2 h. The retinas were dissected as previously described [24]. Samples were incubated with 1% Triton X-100 in PBS for 4 h, and blocked by incubation with 1% BSA. Retinal flatmounts were incubated with rabbit anti-cone arrestin (Millipore, Watford, UK) at 4 °C overnight, followed by Alexa Fluor 594-conjugated donkey anti-rabbit IgG (Life Technologies Ltd., Paisley, UK) for 2 h. Images were obtained by confocal microscopy (Eclipse TE200-U, Nikon UK Ltd., Surry, UK). The Fiji Image-J Software was used for image analysis.

### Immunoblotting

Microglia were lysed in RIPA buffer with protease inhibitor cocktails and phosphatase inhibitors (Sigma-Aldrich). The protein concentration was determined by BCA kit (Thermo Fisher Scientific). Fifty microgram proteins were loaded on a 10% SDS-PAGE gel and transferred to an Immobilon polyvinylidene difluoride membrane (Millipore). The membranes were incubated sequentially with 3–5 ml primary antibodies (Table 2) in 5% dried milk solution in TBS/T overnight at 4 °C. The membranes were washed and incubated with horseradish peroxidase (HRP)-conjugated secondary antibodies (Agilent) for 1 h in the dark at room temperature. The signal was detected by autoradiography using enhanced chemiluminescence (Clarity™ Western ECL Blotting Substrates, BioRad). Densitometric analysis was performed using ImageJ software.

**Table 2:** Antibodies used for western blot

Targets	Primary Antibody (Company; Dilution)	Secondary Antibody (Dilution)
β-actin	Mouse Monoclonal (Santa Cruz, 1:10000)	Polyclonal Rabbit anti-mouse HPR (1:5000)
GLUT1	Rabbit Polyclonal (Thermo Fisher, UK; 1:1000)	Polyclonal Goat anti-Rabbit HPR (1:5000)
GLUT4	Rabbit Polyclonal (Thermo Fisher, UK; 1:1000)	Polyclonal Goat anti-Rabbit HPR (1:5000)
GLUT5	Rabbit Polyclonal (Thermo Fisher, UK; 1:1000)	Polyclonal Goat anti-Rabbit HPR (1:5000)
GLUT9	Rabbit Polyclonal (Millipore Merck, UK; 1:5000)	Polyclonal Goat anti-Rabbit HPR (1:5000)
GLUT10	Rabbit Polyclonal (Thermo Fisher, UK; 1:800)	Polyclonal Goat anti-Rabbit HPR (1:5000)
GLUT12	Rabbit Polyclonal (Antibodies-online; 1:2000)	Polyclonal Goat anti-Rabbit HPR (1:5000)

### Statistical analyses

Data were processed and analyzed by GraphPad Prism Software and presented as mean ± SEM. Comparisons between more than two groups were conducted using one-way ANOVA followed Dunnett’s Multiple Comparison Tests. Probability values of < 0.05 were considered significant.

## Results

### Characteristics of a newly established microglial cell line B6M7 cells

**Fig. 1:**
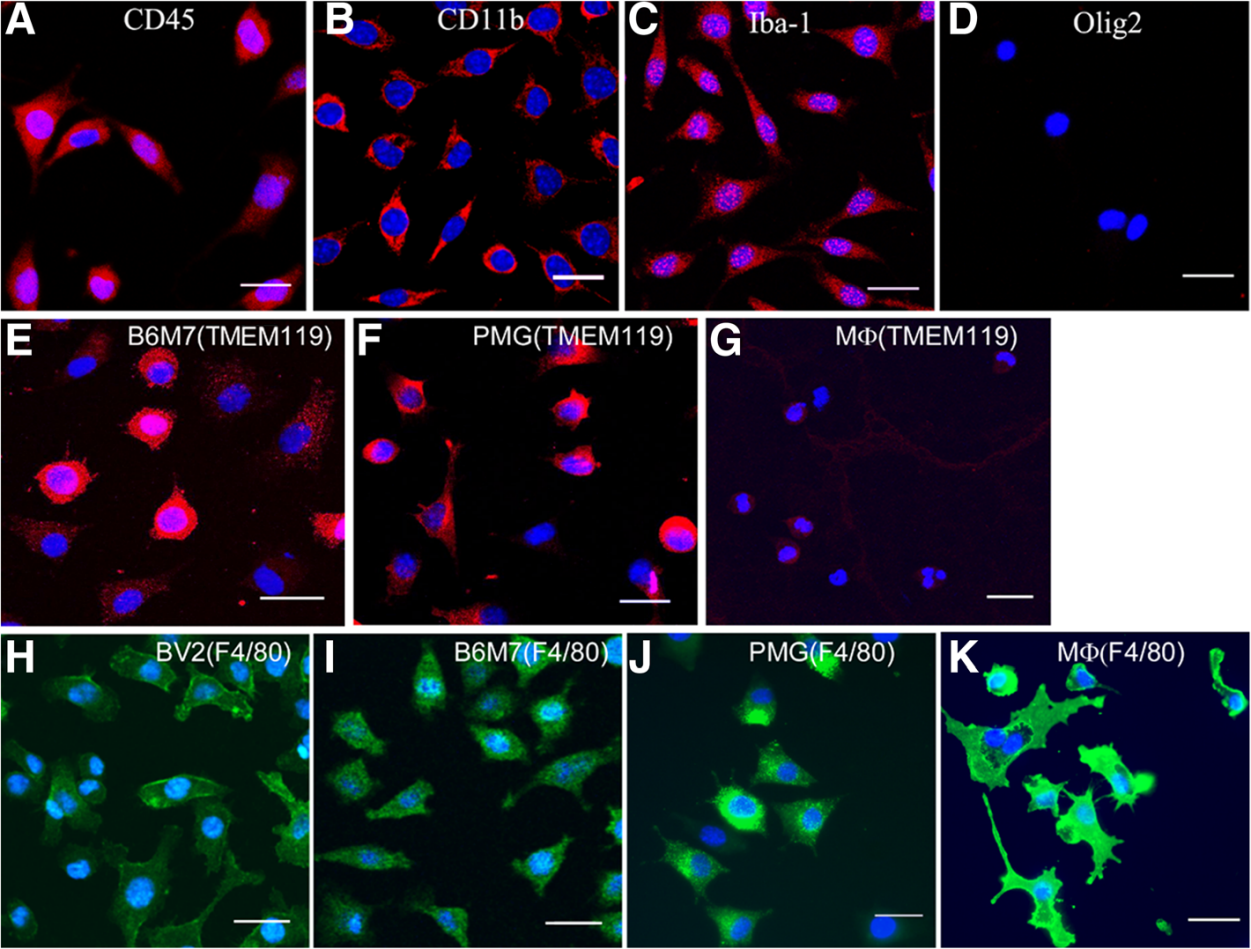
Immunocytochemistry of B6M7 cells. **a**-**d** B6M7 cells were stained for CD45 (**a**), CD11b (**b**), Iba1 (**c**), and Olig2 (**d**). **e**-**g** TMEM119 expression (red) in B6M7, primary microglia (PMG) and peritoneal macrophages (MФ). (**h**-**k**) F4/80 expression (green) in BV2, B6M7, PMG and peritoneal macrophages. Cell nuclei were stained with DAPI. All samples were examined by confocal microscopy. Scale bar = 25 μm

Immunocytochemistry showed that the B6M7 cells express CD45, CD11b, Iba-1, TMEM119, but not Olig2 (Fig. 1a-e). The majority of B6M7 and primary microglial (PMG) cells were strong positive for TMEM119 (Fig. 1e, f), whereas negligible levels of TMEM119 were detected in peritoneal macrophages (Fig. 1g). The macrophage marker F4/80 was strongly expressed by peritoneal macrophages, but only weakly expressed by microglia including BV2, B6M7 cells and PMG (Fig. 1h-k). Furthermore, B6M7 and BV2 cells, but not peritoneal macrophages, showed a strong response to ATP-induced chemotaxis (Additional file 1: Figure S1). The results suggest that B6M7 cells maintain characteristics of microglia.

### Glucose transporter expression by microglial cells

**Fig. 2:**
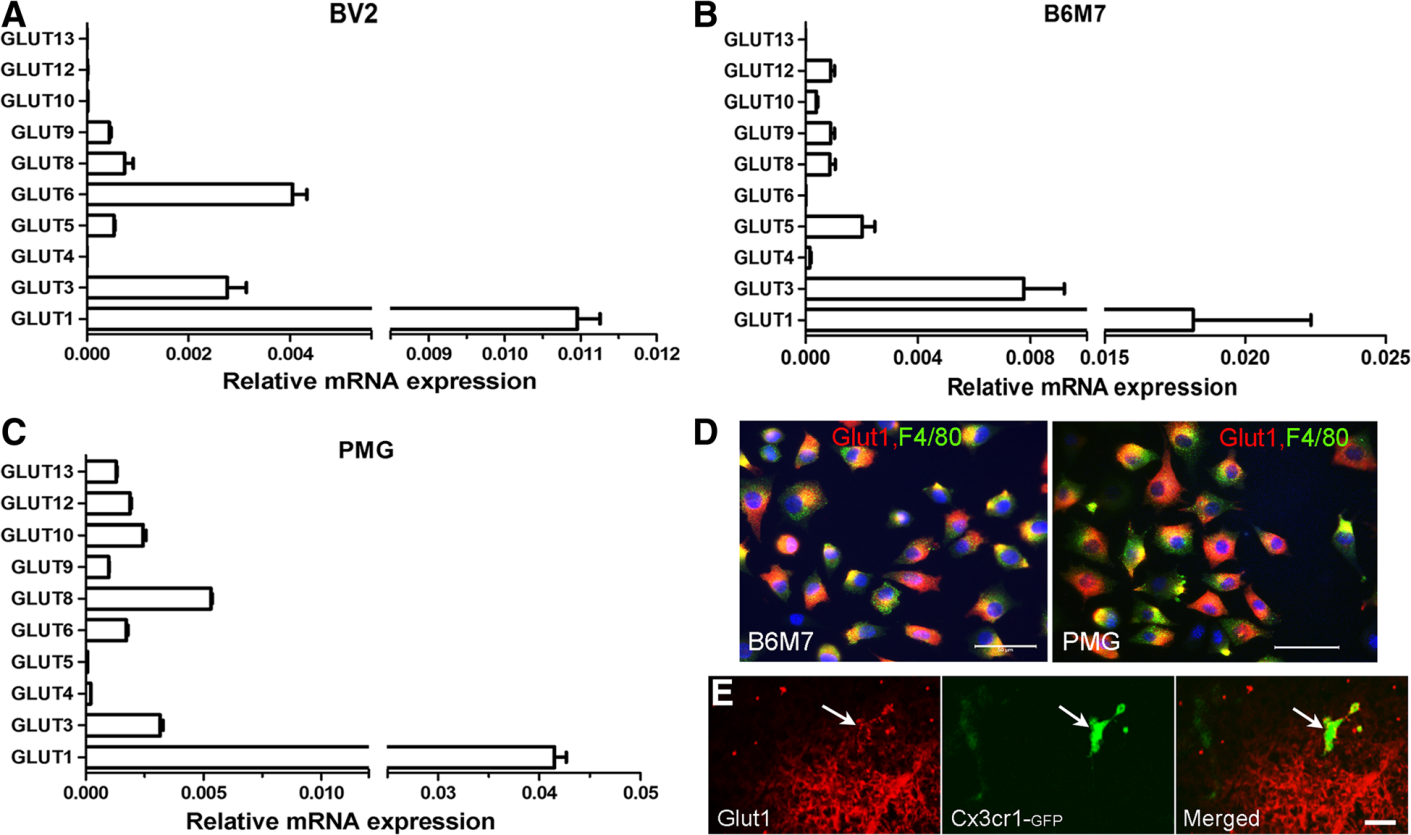
GLUT expression in microglial cells. Quantitative RT-PCR analysis of GLUT-1, 3, 4, 5, 6, 7, 8, 9, 10, 12 and 13 in BV2 (**a**), B6M7 (**b**) and primary microglial (PMG, **c**) cells. Data were expressed as relative expression of GLUTs against β-actin. Mean ± SEM, *n* = 6. **d** Confocal images showing GLUT1 (red) and F4/80 expression in B6M7 and primary microglia. Scal bar = 50 μm. **e** Retinal section from CX3CR1^GFP/+^ mice was stained for GLUT1 (red) and imaged by confocal microscopy. Arrow indictes a GFP positive microglia expressing GLUT1. Scale bar = 20 μm

To understand which glucose transporters control glucose uptake in microglia, we examined the expression of different GLUTs in BV2, B6M7, and primary microglia. Initial conventional PCR detected the mRNA of GLUT1, 3, 4, 5, 6, 8, 9, 10, 12, and 13 in primary microglial cells (data not shown). Real-time RT-PCR was then employed to determine relative expression levels of GLUTs in different microglial cells. Among the 10 detected GLUTs, GLUT1 had the highest levels of expression in BV2 (Fig. 2a), B6M7 (Fig. 2b) and primary microglia (PMG, Fig. 2c) cells. GLUT3 was also expressed at high levels in all types of microglial cells (Fig. 2a-c). The expression levels of other GLUTs vary in different types of microglial cells. For example, GLUT5 was expressed at high levels in B6M7 cells (Fig. 2b), but at low levels in primary microglia (Fig. 2c). The expression of GLUT1 in B6M7, primary microglia (Fig. 2d) and in vivo retinal microglia (Fig. 2e) was further confirmed by immunostaining.

### Increased GLUT-1 expression in inflammatory microglia

**Fig. 3:**
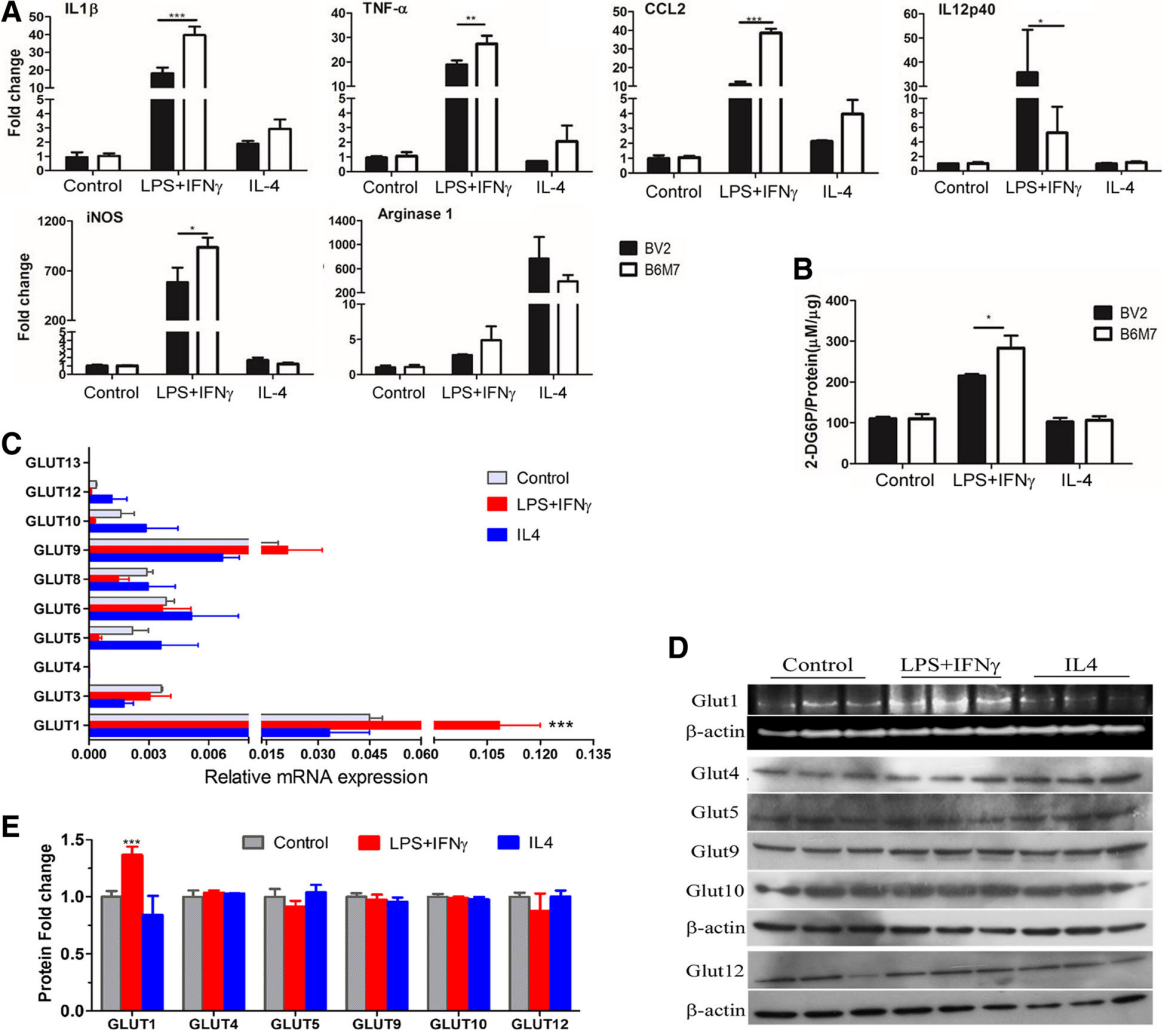
Changes in glucose uptake and GLUT expression during microglial activation. BV2 and B6M7 cells were untreated or treated with LPS + IFNγ or IL-4 for 24 h. Cells were then processed for real-time RT-PCR (**a**, **c**), glucose uptake (**b**) or Western blot (**d**). **a** The expression of inflammatory mediators in naïve, LPS + IFNγ or IL-4 treated BV2 and B6M7 cells. *N* = 3, *, *P* < 0.05; **, *P* < 0.01, ***P < 0.001. **b** Glucose uptake in non-treated control, LPS + IFNγ or IL-4 treated BV2 and B6M7 cells. N = 3, *, *P* < 0.05. **c** mRNA expression of different GLUTs in control, LPS + IFNγ or IL-4 treated B6M7 cells. *N* = 3, ***, *P* < 0.001 compared to naïve microglia. **d** Western blot showing GLUT1, 4, 5, 9, 10, 12 in control (naïve), LPS + IFNγ treated, and IL-4 treated B6M7 cells. β-actin was used as housekeeping control. **e** Densitometric analysis showing GLUT expression related to control in LPS + IFNγ or IL-4 treated B6M7 cells. N = 3, ***, *P* < 0.001 compared to naïve microglia

LPS + IFNγ treatment significantly upregulated the expression of IL-1β, TNFα, CCL2, IL-12p40 and iNOS, whereas IL-4 treatment increased the expression of Arginase-1 in BV2 and B6M7 cells (Fig. 3a). In general, B6M7 cells had higher levels of inflammatory gene expression following LPS + IFNγ stimulation compared to BV2 cells (apart from IL-12p40, Fig. 3a). To understand how glucose metabolism is related to microglial activation, glucose uptake was measured in naïve and activated microglial cells. M(LPS + IFNγ) microglia consumed significantly higher amounts of glucose than naïve and M(IL-4) microglia (Fig. 3b). In line with higher levels of inflammatory cytokine expression, glucose uptake was also significantly higher in B6M7 M(LPS + IFNγ) cells compared with BV2 M(LPS + IFNγ) cells (Fig. 3b). Real-time RT-PCR showed that only the expression of GLUT1 was significantly increased following LPS + IFNγ stimulation (Fig. 3c). None of the GLUTs were affected by IL-4 treatment (Fig. 3c). The upregulation of GLUT1 by LPS + IFNγ treatment was further confirmed at the protein level by Western Blot (Fig. 3d-e).

### Bioenergetic profiles in active microglia

**Fig. 4:**
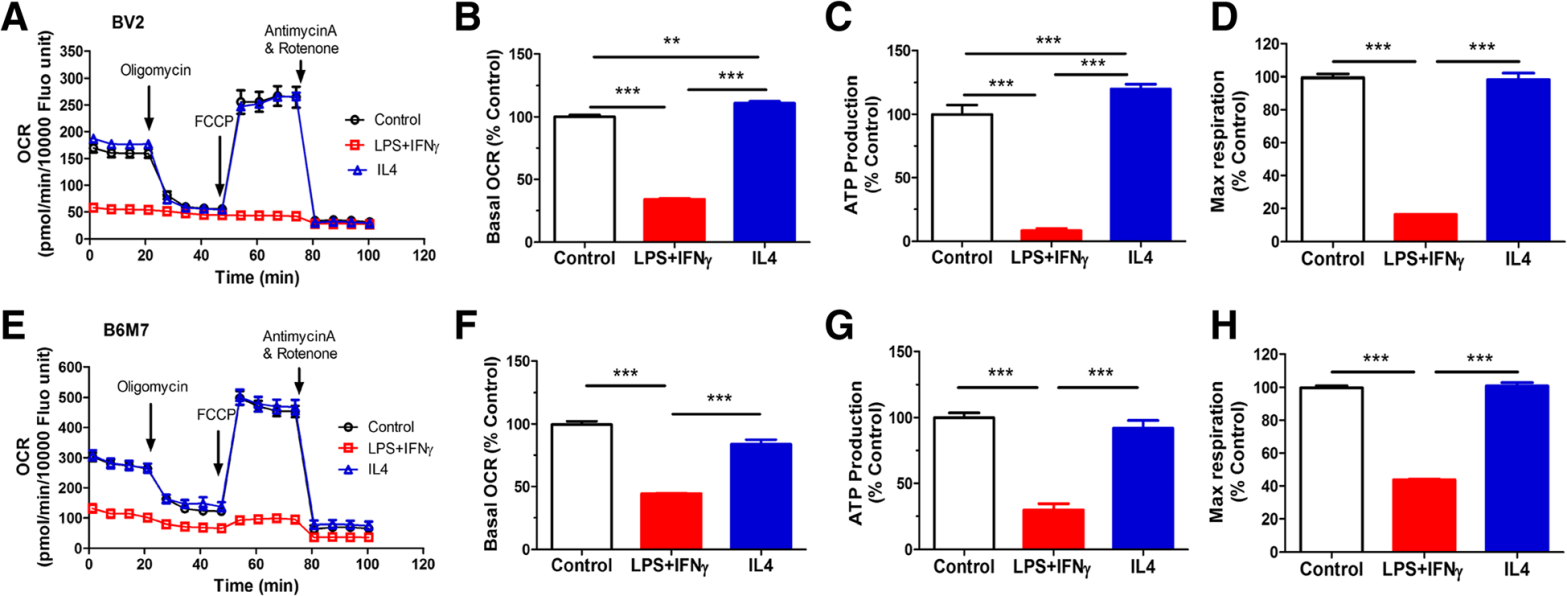
Mitochondrial respiration in different types of microglial cells. BV2 and B6M7 cells were untreated (naïve), or treated with LPS + IFNγor IL-4 for 24 h. The cells were then subjected to Mito Stress Test. The OCR was measured sequentially under basal conditions, following inhibition of ATP synthase (with oligomycin), uncoupling the electron transporter chain (ETC) with FCCP, and blocking complex I and III with rotenone and antimycin A. **a**, **e** Representative OCR profiles of BV2 (**a**) and B6M7 (**e**) naïve, LPS + IFNγ- or IL-4-treated cells. **b**, **f** Changes in basal OCR in LPS + IFNγ treated and IL-4 treated cells compared to naïve microglia. **c**, **g** Changes in ATP production in LPS + IFNγ- and IL-4-treated microglial cells compared to naïve microglia. **d**, **h** Changes in maximal respiration in LPS + IFNγ- and IL-4-treated microglial cells compared to naïve microglia. Mean ± SEM, *n* = 3 repeated experiments, **P < 0.01, ***P < 0.001

**Fig. 5:**
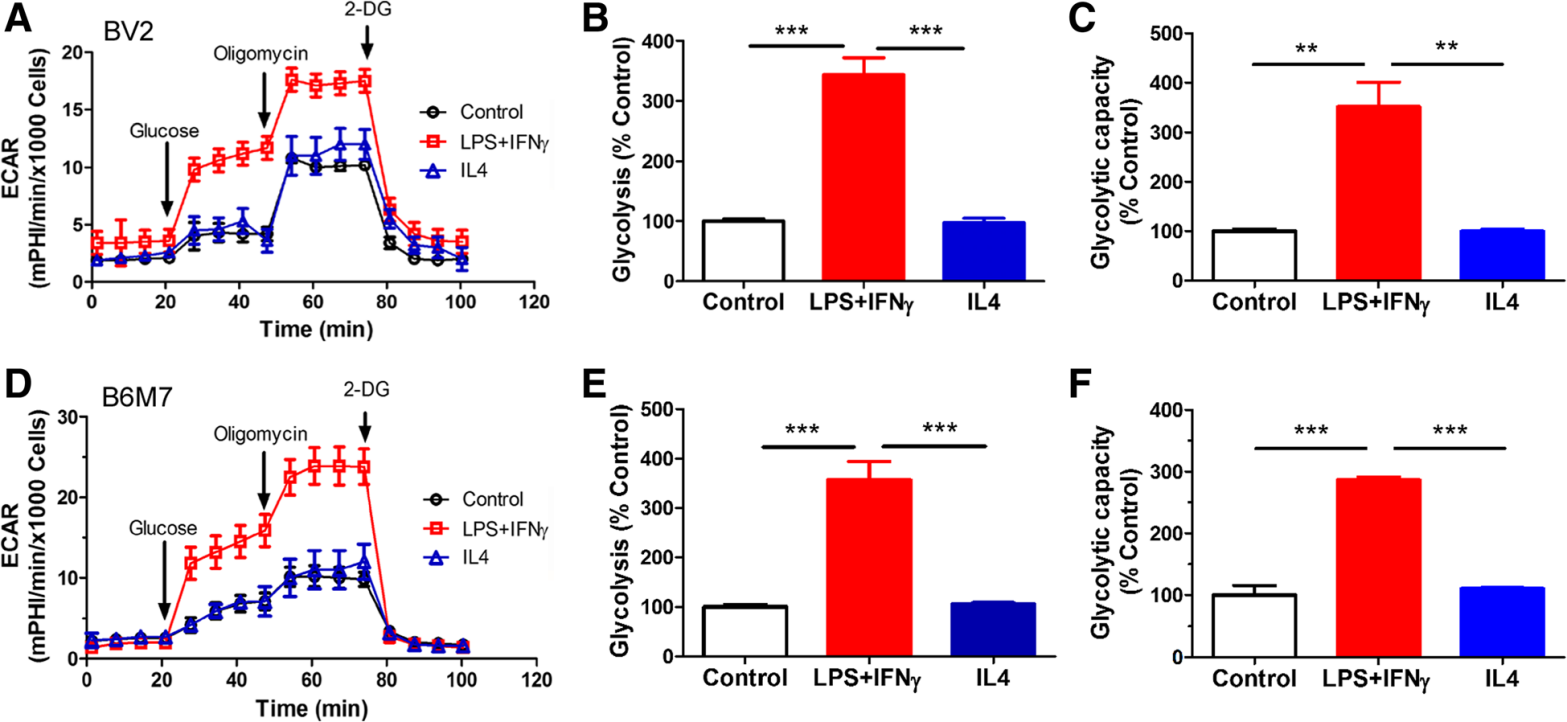
Glucose metabolism in different types of microglial cells. BV2 and B6M7 cells were untreated (control), or treated with LPS + IFNγ or IL-4 for 24 h. **a**, **d** Representative ECAR profiles of BV2 (**a**) and B6M7 (**d**) cells following glucose, oligomycin and 2-DG treatments in the Glycolysis Stress assay. **b**, **e** Changes in basal ECAR in LPS + IFNγ treated and IL-4 treated microglial cells compared to naïve microglia. **c**, **f** Changes in glycolytic capacity in LPS + IFNγ treated and IL-4 treated cells compared to naïve microglia. Mean ± SEM, n = 3 repeated experiments, **P < 0.01, ***P < 0.001

The bioenergetic profiles of microglial cells were examined using the Seahorse XFe96 Analyser. The basal OCR, ATP production and maximal respiration of M(LPS + IFNγ) microglia was significantly reduced in both BV2 (Fig. 4a-d) and B6M7 (Fig. 4e-h) cells compared to naïve microglia. IL-4 treatment slightly increased the basal OCR and ATP production in BV2 (Fig. 4b, c) but not in B6M7 cells (Fig. 4f, g). The glycolysis and glycolytic capacity were significantly increased following LPS + IFNγ but not IL-4 stimulation in both BV2 (Fig. 5a-c) and B6M7 cells (Fig. 5d-f). Our results suggest that M(LPS + IFNγ) microglia are fueled predominately by glycolysis rather than mitochondrial respiratory phosphorylation.

### The role of GLUT1 in microglial metabolism

**Fig. 6:**
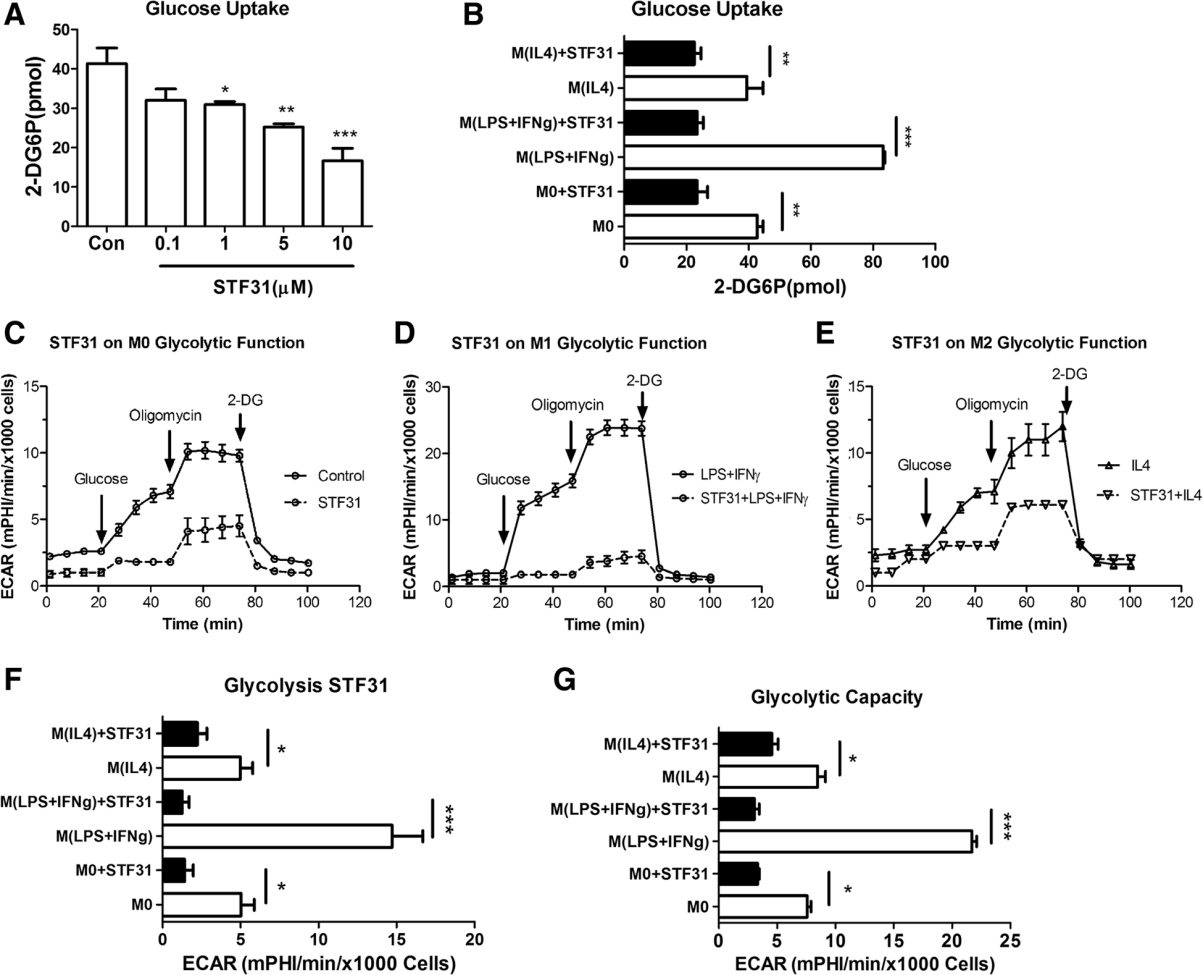
The effect of STF31 on microglial glucose metabolism. **a** Naïve B6M7 cells were treated with different concentrations of STF31 for 24 h. Glucose uptake was then conducted. n = 3, *, *P* < 0.05; **, *P* < 0.01; ***, *P* < 0.001, One-way ANOVA with Dunnett’s Multiple post-test. **b** Glucose uptake in naïve (M0), LPS + IFNγ treated and IL-4 treated microglial cells with or without 5 μM STF31 pre-treatment for 24 h. n = 3, **, *P* < 0.01; ***, *P* < 0.001, two-way ANOVA with Bonferroni post-test. **c**-**e** Representative ECAR profiles in naïve (**c**), LPS + IFNγ treated (**d**), and IL-4 treated (**e**) microglia with or without 5 μM STF31. **f** The effect of STF31 on glycolysis of M0, M(LPS + IFNγ), and M(IL-4) B6M7 microglial cells. **g** The effect of STF31 on glycolytic capacity of naive, LPS + IFNγ treated and IL-4 treated B6M7 cells. Mean ± SEM, n = 3 repeated experiments, *P < 0.05; **P < 0.01; ***P < 0.001

Having shown that M(LPS + IFNγ) microglia are fueled by the glycolytic pathway and GLUT1 is highly expressed in these cells, we then examined the effect of a GLUT1 specific inhibitor STF31 [25] on microglial activation. STF31 dose-dependently suppressed glucose uptake in B6M7 cells (Fig. 6a) without affecting cell viability (Additional file 1: Figure S2A, B). 5 μM of STF31 significantly reduced glucose uptake in naïve, M(LPS + IFNγ) and M(IL-4) microglial cells (Fig. 6b). The expression of GLUT1 and other GLUTs was not affected by STF31 treatment (Additional file 1: Figure S2C-E). This concentration of STF31 was, therefore, used in the rest of the in vitro studies.

Glycolysis stress test in naïve (Fig. 6c), M(LPS + IFNγ) (Fig. 6d) and M(IL-4) (Fig. 6e) cells showed that STF31 (5 μM) significantly reduced glycolysis (Fig. 6f) and glycolytic capacity (Fig. 6g), suggesting shutting-down of the glycolytic pathway by STF31.

**Fig. 7:**
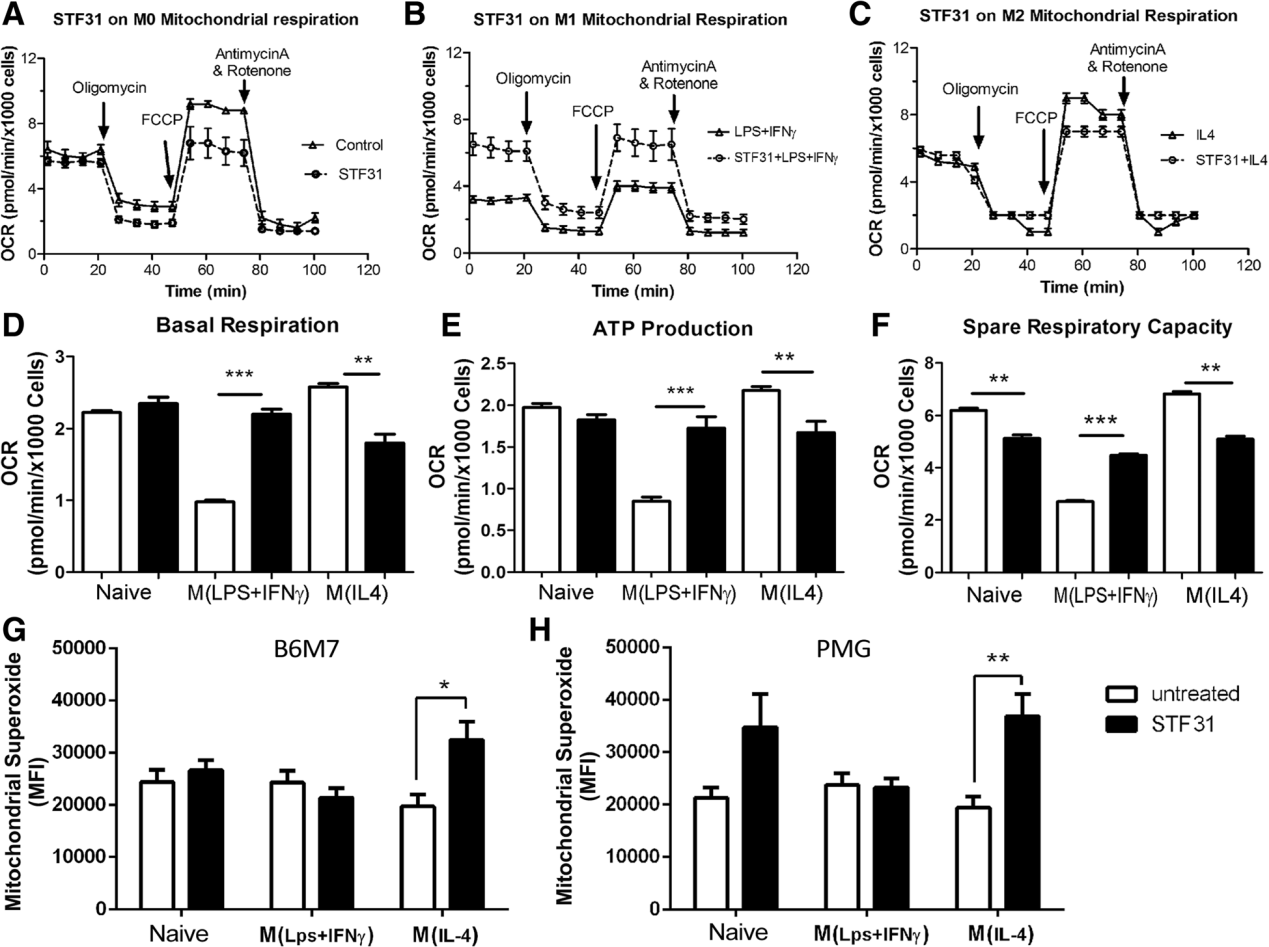
The effect of STF31 on microglial mitochondrial respiration. B6M7 cells were incubated with 5 μM STF31, and then stimulated with LPS + IFNγ or IL-4. The cells were then subjected to Mito Stress assay (**a**-**f**) or MitoSOX Red assay (**g**, **h**). **a**-**c** Representative OCR profiles in naïve (**a**), M(LPS + IFNγ) (**b**), and M(IL-4) (**c**) microglia with or without 5 μM STF31. **d** The effect of STF31 on microglial basal respiration of naïve, M(LPS + IFNγ), and M(IL-4) microglial cells. **e** The effect of STF31 on ATP production of naive, M(LPS + IFNγ), and M(IL-4) microglial cells. **f** The effect of STF31 on spare respiratory capacity of naive, M(LPS + IFNγ), and M(IL-4) microglial cells. **g**, **h** The effect of STF31 on the production of mitochondrial superoxidate in naive, LPS + IFNγ, IL-4 treated B6M7 (**g**) and primary microglia (PMG, **h**). Mean ± SEM, n = 3 repeated experiments, *, *P* < 0.05; **P < 0.01; ***P < 0.001

The basal OCR and ATP production was not affected by STF31 in naïve microglia in Mito stress assay (Fig. 7a, d-e). However, the spare respiratory capacity (SRC) was significantly reduced following STF31 treatment (Fig. 7f), suggesting that glucose is needed for maximal respiration but not for basal respiration and ATP production in naïve microglia.

In M(LPS + IFNγ) microglia, the treatment of STF31 significantly increased basal OCR, ATP production, and SRC (Fig. 7b, d-f), suggesting re-programming of the metabolic pathway towards mitochondrial respiration. Interestingly, STF31 treatment significantly decreased basal OCR, ATP production and SRC in M(IL-4) microglia (Fig. 7c, d-f), indicating that mitochondrial respiration in these cells is fueled partially by glucose.

Mitochondrial superoxide production (measured by MitoSOX Red) did not change following LPS + IFNγ or IL-4 stimulation in B6M7 and primary microglia (Fig. 7g, h). Blocking glucose uptake by STF31 significantly increased mitochondrial superoxide in M(IL-4) microglia (Fig. 7g, h), suggesting that Glut-1 mediated glucose metabolism is important to maintain mitochondrial membrane potential in these cells.

Taken together, our results suggest that GLUT1 critically control glucose uptake in microglial cells and blocking GLUT1 with STF31 switches the bioenergetic pathway from glycolysis to OXPHOX in M(LPS + IFNγ) microglia but reduces OXPHOX in M(IL-4) microglia.

### The effect of STF31 on microglial phagocytosis and activation

**Fig. 8:**
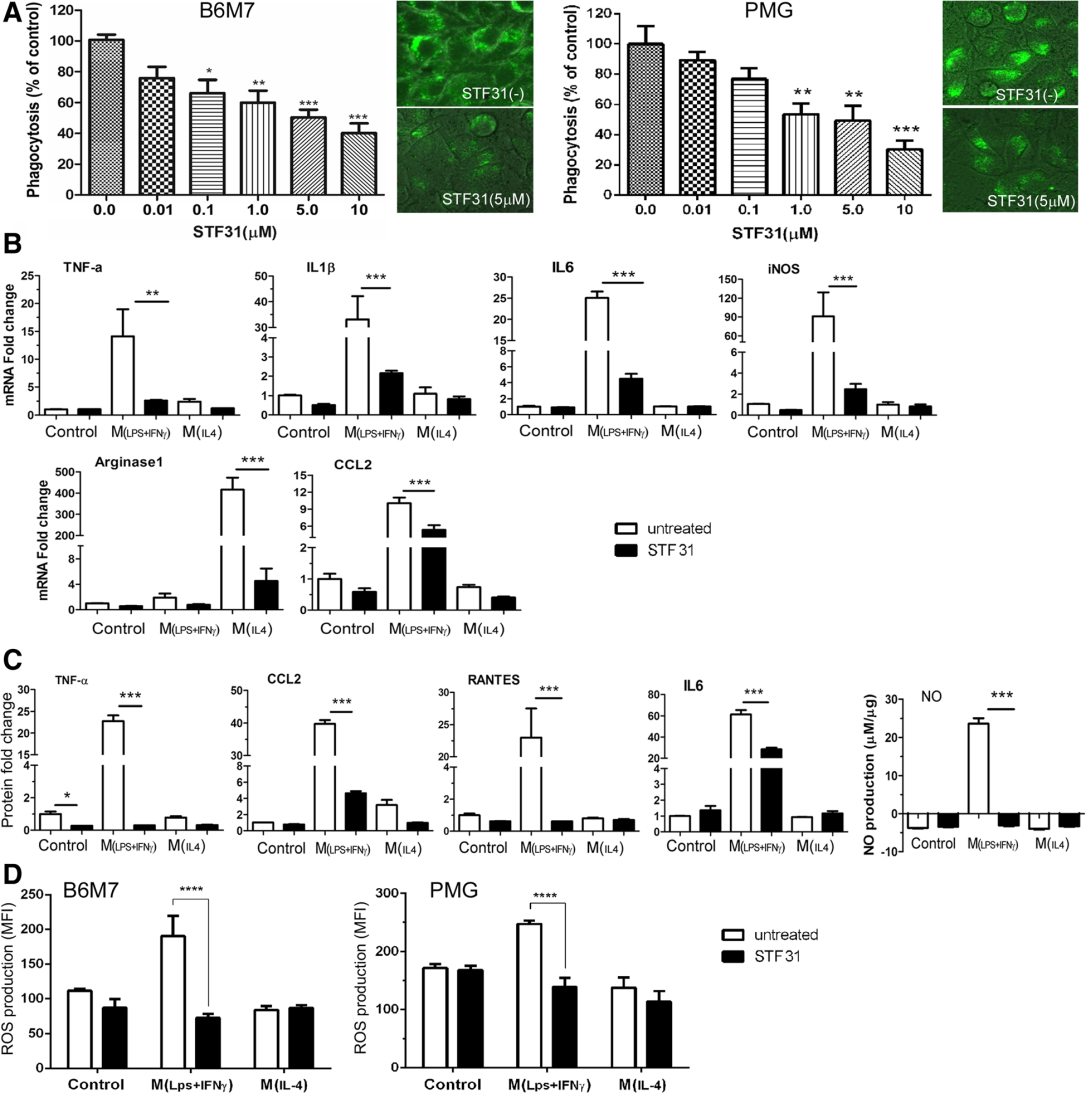
The effect of STF31 on microglial phagocytosis, cytokine expression/secretion and intracellular reactive oxygen spececis production. **a** B6M7 cells and primary microglia (PMG) were incubated with 5 μM STF31 for 24 h. Phagocytosis was performed using the pHrodo Green *S. aureus* BioParticles. Inserted images showing green fluorescence from phagocytosed *S. aureus* bioparticles in different groups of cells. Mean ± SEM, n = 3 (repeated experiments), *P < 0.05; **P < 0.01; ***P < 0.001. One-way ANOVA with Tukey’s Multiple Comparison Test. **b**-**d** B6M7 cells were incubated with 5 μM STF31 for 24 h followed by LPS + IFNγ or IL-4 stimulation. The mRNA expression of TNFα, IL-β, IL-6, iNOS, CCL2 and arginase-1 was measured by real-time RT-PCR (**b**). **c** The production of TNFα, CCL2, RANTES, and IL-6 was measured by CBA, and NO production was determined by Griess Assay. **d** The effect of STF31 on intracellular reactive oxygen species (ROS) production in naive, LPS + IFNγ, IL-4 treated B6M7 and primary microglia (PMG) meased by CellRox Green assay. Mean ± SEM, n = 3 repeated experiments, **P < 0.01; ***P < 0.001; ****P < 0.0001

The phagocytosis of *S.aureus* bioparticles by B6M7 and primary microglia was dose-dependently suppressed by STF31 (Fig. 8a). Real time RT-PCR showed that STF31 treatment significantly reduced the expression of TNFα, IL-1β, IL-6, iNOS, and CCL2 in M(LPS + IFNγ) cells (Fig. 8b). The production of TNFα, CCL2, RANTES, IL-6, and NO was also significantly reduced following STF31 treatment (Fig. 8c). Interestingly, the treatment also suppressed IL-4-induced arginase-1 expression (Fig. 8b).

Intracellular ROS was significantly increased following LPS + IFNγ treatment in both B6M7 and primary microglia, and the increment was prevented by STF31 treatment (Fig. 8d).

### In vivo effect of STF31 on microglial activation and retinal degeneration

Uncontrolled microglial activation is known to contribute to photoreceptor death in the mouse model of light-induced retinal degeneration [26]. Therefore, we further tested the effect of STF31 on microglial activation in this model. Intraperitoneal injection of STF31 (10 mg/kg, for 5 days) in normal mice did not affect their body weight, behavior, and ERG responses (Additional file 1: Figure S3A, B). The treatment did not induce any retinal cell death (Additional file 1: Figure S3C).

**Fig. 9:**
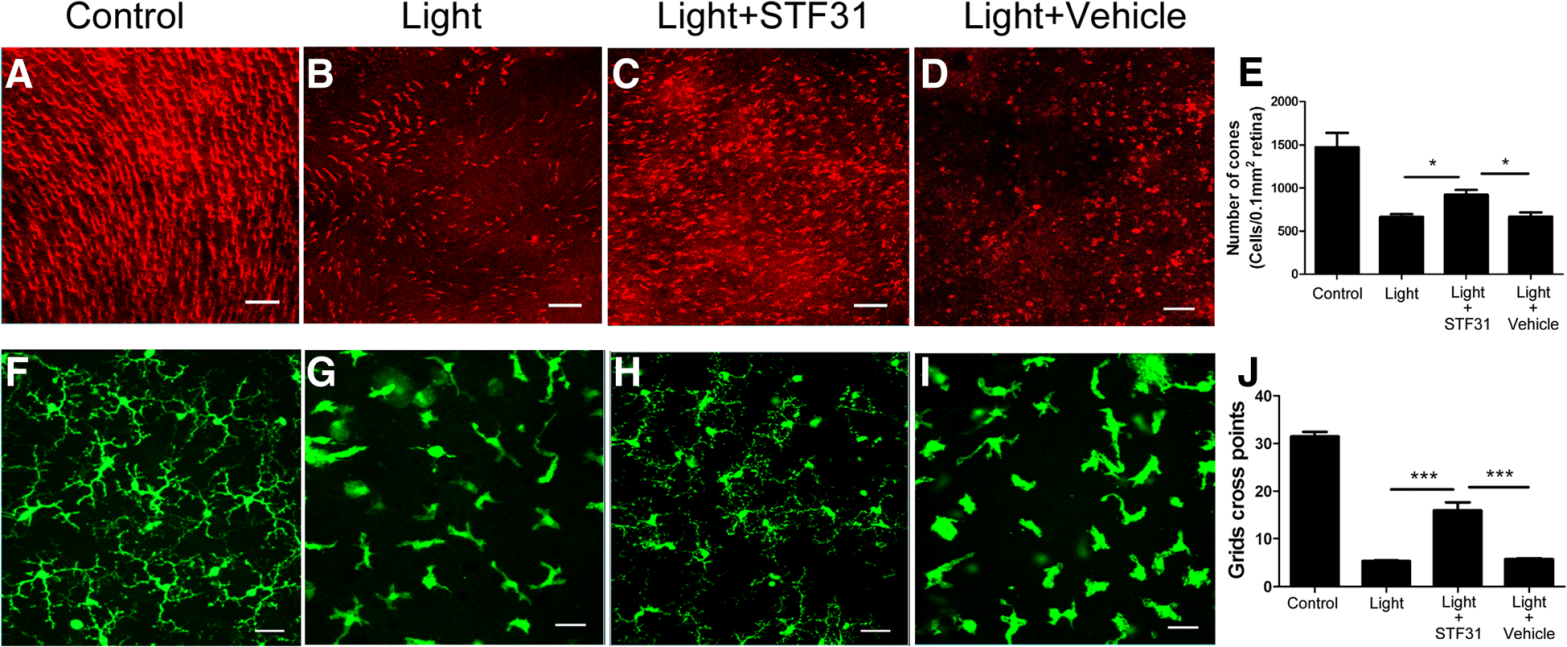
The effect of STF31 on light-induced photoreceptor degeneration and microglial activation. *CX3CR1*^*gfp/+*^ mice were exposed to 50,000 lx focal white light for 10 min with daily STF31 or vehicle (DMSO) treatment. Eyes were collected 4 days after light exposure. **a**-**d** Representative confocal images showing cone arrestin-positive cells in control (**a**), light-exposed (**b**), light + STF31 (**c**) and light + DMSO (**d**) mice. **e** Graph showing the number of cone photoreceptors in different groups of mice. **f**-**i** Representative confocal images showing GFP-positive retinal microglia in normal (**f**), light-exposed (**g**), light + STF31 (**h**) and light + DMSO (I) mice. **j** Graph showing the average number of cross points of microglia in different groups of mice. Mean ± SEM. A minimum of 6 images was taken from each retina. Full control, *n* = 4 mice, *n* = 6 mice in other groups. * *P* < 0.05; ***P < 0.001. Scale bar: 50 μm

Massive photoreceptor loss (Fig. 9a-b, e) and microglial activation (Fig. 9f-g, j) were observed 4 days after light exposure. STF31 treatment improved photoreceptor survival and reduced microglial activation (Fig. 9). The treatment with vehicle (DMSO) did not show any protective effect (Fig. 9). SD-OCT examination showed improvement in both retinal structure and thickness, particularly the outer retinal layers (from ONL to IS/OS, Additional file 1: Figure S4E, G) following STF31 treatment (Additional file 1: Figure S4).The treatment did not significantly improve the thickness of the inner retina (from NFL/GL to OPL, Additional file 1: Figure S4E, F).

## Discussion

Glucose is essential for microglial survival and function. A previous study has shown that glucose depletion led to 74% microglia death within 30 min and 98% after 48 h [27]. Here, we show that glucose uptake in microglia is mediated predominately by GLUT1. Importantly, we show that glycolysis in inflammatory M(LPS + IFNγ) microglia also relays on GLUT1-mediated glucose uptake, and blocking GLUT1 re-programmed proinflammatory microglia from glycolysis to OXPHOX, suppressed microglial activation and reduced light-induced retinal degeneration. Our data also show that GLUT1-mediated glucose metabolism is critically involved in mitochondrial respiration in M(IL-4) microglia. Our results highlight the importance of GLUT1-mediated glycolysis in microglial function under pathophysiological conditions.

Naïve microglia are fueled by both glycolysis and mitochondrial oxidative phosphorylation. Previous studies have shown that microglia express GLUT3, GLUT4 [28], and GLUT5 [29, 30]. GLUT1 was reported to be expressed in oligodendrocytes and astrocytes but not microglia [31]. In the current study, we detected 13 GLUTs in murine primary microglia, BV2, and a newly established murine microglial cell line B6M7 cells. Surprisingly, we found that GLUT1 was expressed at the highest level among other GLUTs, and the expression of GLUT1 was further enhanced following LPS + IFNγ stimulation. This is in line with the metabolic re-programming of the cells towards anaerobic glycolysis. The expression of other GLUTs was not affected by LPS + IFNγ treatment. Our results suggest that GLUT1 critically controls glucose uptake and the glycolytic pathway in microglial cells, particularly under inflammatory conditions.

Phagocytosis is important for microglia to maintain CNS homeostasis. We show that blocking GLUT1-mediated glucose uptake reduced glycolysis (Fig. 6) and suppressed phagocytosis (Fig. 8a). Our results suggest that GLUT1-mediated glycolysis is critically involved in microglial phagocytosis. This is in line with our recent observation in the phagocytic function of peritoneal macrophages [32]. Blocking GLUT1 in naïve microglia did not affect mitochondrial basal respiration and ATP production, but significantly reduced SRC (Fig. 7f). SRC is important for cells to deal with stress conditions when additional energy is required. Our results suggest that GLUT1-dependent glycolysis may provide reserved energy to microglia under stress conditions for survival and function.

Microglial activation is a necessary and beneficial response to CNS insults. However, uncontrolled microglial activation contributes critically to the pathogenesis of various neurodegenerative diseases [33] through the release of excessive amount of inflammatory mediators. Recent studies have shown that M1-type proinflammatory microglia are fueled by anaerobic glycolysis [18]. Previous studies have shown that the glycolysis pathway provides essential building blocks such as amino acids and fatty acids for the assembling of inflammatory mediators in macrophages [34–36] and effector T helper cells [37–39]. Our data and that of others [18] suggest that inflammatory microglia are also fueled by the glycolytic pathway.

A recent study using dual tracer positron emission tomography has shown that following cerebral ischemia, neurons and astrocytes died from oxygen deficiency but immune cells metabolized glucose non-oxidatively [40], suggesting that they are likely supported by anaerobic glycolysis at the ischemic inflammatory site. The immune cells at the lesion sites may consist of resident microglia and infiltrating leukocytes. Shifting immune cell metabolism towards anaerobic glycolysis in an environment in which the supply of oxygen and nutrients is restricted requires the upregulation of the facilitators of glucose transport. Our results suggest that inflammatory microglia upregulate GLUT1 to facilitate glucose uptake.

Using STF31, we show that when GLUT1-mediated glucose uptake is blocked in inflammatory microglia, the metabolic pathway can be further re-programmed towards OXPHOX. Consequently, the expression of inflammatory genes and the production of inflammatory mediators are reduced. It is important to note that although STF31 dose-dependently reduced glucose uptake in microglia, it did not completely block glucose uptake (Fig. 6a). 5 μm STF31 reduced glucose uptake from 85 pmol to 25 pmol in M(LPS + IFNγ) microglia (Fig. 6b), and glycolysis was not diminished (Fig. 6c-e). The results suggest that apart from GLUT1, other GLUTs also facilitate glucose uptake in microglia. This may explain why microglia did not undergo cell death after STF31 treatment as seen in some tumor cells [41].

Another important observation of this study is the requirement of glucose in mitochondrial OXPHOX in alternatively active M(IL-4) microglia. Previous studies have shown that the M(IL-4) microglia are supported by an intact tricarboxylic acid (TCA) cycle and enhanced mitochondrial OXPHOX [18]. However, the fuel source of OXPHOX in M(IL-4) microglia remains elusive. We show that when glucose uptake is restricted by STF31, mitochondrial basal respiration, ATP production and SRC were all significantly reduced, whereas mitochondrial superoxidate is increased in M(IL-4) microglia. Previously, fatty acids were shown to support mitochondrial oxidative metabolism in M(IL-4) macrophages [42]. Recent studies suggest that glucose can also fuel OXPHOX in M(IL-4) macrophages [43–45]. Our results suggest that mitochondrial respiration in M(IL-4) microglia is at least partially fueled by glucose.

The newly established microglial cell line B6M7 cells were used in this study. The cells express high levels of TMEM119 (a microglial-specific marker) but low levels of F4/80 (a typical macrophage marker). ATP is known to induce chemotaxis in microglia but not macrophage through Gi/o-coupled P2Y receptors [46]. The B6M7 cells, but not peritoneal macrophages had a strong chemotactic response to ATP stimulation. Our results suggest that B6M7 cells phenotypically and functionally resemble microglia but not macrophages. Lineage tracing study will help to confirm the true identity of the cells. The B6M7 cells and BV2 cells have some functional differences. For example, ATP-induced chemotaxis is significantly higher in B6M7 cells than that in BV2 cells. The LPS + IFNγ-induced inflammatory gene expression was also significantly higher in B6M7 cells than that in BV2 cells. The B6M7 cells could be an useful additional resource in neuroimmunology studies.

## Conclusion

Glucose uptake in microglia is dominated by GLUT1. Under inflammatory conditions, microglia further upregulate GLUT1 to facilitate glucose uptake and promote anaerobic glycolysis. Blocking GLUT1 is an effective approach to re-program the metabolic pathway and control microglial activation and neurodegeneration. As glucose uptake in neurons is mediated predominately by GLUT3 [20], blocking GLUT1 may not only suppress microglial activation, but also increase available nutrients to neurons, particularly in the inflamed ischemic CNS when glucose supply is restricted. Since GLUT1 is expressed by many other cells (e.g., astrocytes, Muller cells, photoreceptors, endothelial cells) in the retina and it critically controls glucose uptake to CNS parenchyma at the blood-brain-barrier [20], cell-specific inhibition of GLUT1 (e.g., via CX3CR1 or CFSR promoters) would be an ideal approach to control microglial activation and neuroinflammation.

## Additional file

Additional file 1: **Figure S1.** ATP-induced microglial migration using the transwell assay. B6M7, BV2 cells and peritoneal macrophages (3,000/well) were seeded into the upper chamber in a trans-well culture system for 16h. 100 μM ATP was then added into the bottom chamber. 3 hours later, cell numbers in bottom chambers were counted. Mean ±SEM, N=6 ***P<0.001. **Figure S2.** The effect of STF31 on microglial cells. (A) B6M7 cells were treated with different concentrations of STF31 and cell viability was assessed by AlamarBlue 24 h and 48 h after treatment. N=3. (B) Representative images and quantifications of TUNEL assay in naïve, M(LPS+IFNγ), and M(IL-4) microglia with or without 5 μM STF31. Arrow shows a TUNEL-positive cell. Scale bar = 50 μm. N=3. (C, D) RT-PCR (C) and Western Blot (D) showing the expression of GLUT1 in B6M7, with or without 5 μM STF31. (E) RT-PCR showing the expression of GLUTs in B6M7 microglial cells, with and without 5 μM STF31. RT-PCR and western blot data were expressed as relative expression against β-actin (*n*=3). Mean ±SEM. **Figure S3.** The effect of STF31 on mouse retina. Mice were injected intraperitoneally with STF31 (10 mg/kg) for 5 days. Four mice were used. (A) Representative ERG responses at day 0 (before the onset of STF31 treatment) and day 4. (B) Quantitative analysis of a-wave and b-wave amplitudes, before (day 0) and after (day 4) STF31 treatment. (C, D) Representative images (C) and quantifications (D) of TUNEL assay in control and STF31-treated mice. Scale bar = 25 μm. **Figure S4.** The effect of STF31 on retinal thickness in light-induced retinal degeneration. CX3CR1^gfp/+^ mice were exposed to 50,000 lux focal white light for 10 minutes with daily STF31 or vehicle (DMSO) treatment. (A-D) Representative images from each group of mice. (E) Bar graph showing the average thickness of the entire neuronal retina (from nerve fiber/ganglion layer to inner/outer segments layer, illustrated in A) in each group. (F) Bar graph showing the thickness of the inner retina (from nerve fiber/ganglion layer to inner plexiform layer, illustrated in A). (G) Bar graph showing the thickness of the outer retina (from outer nuclear to inner/outer segments, illustrated in A). Full control: N=4 mice, N=6 mice in other groups. **P < 0.01; ***P < 0.001. (DOCX 1656 kb)

## Abbreviations

2DG: 2-deoxyglucose
ATP: Adenosine triphosphate
CNS: Central nervous system
DMSO: Dimethyl sulfoxide
ECAR: Extracellular Acidification Rate
ERG: Electroretinography
FCS: Fetal calf serum
GLUT: Glucose transporter
IFNγ: Interferon gamma
IL-4: Interleukin 4
LPS: Lipopolysaccharide
NO: Nitrite oxide
OCR: Oxygen Consumption Rate
OXPHOX: Oxidative phosphorylation
ROS: Reactive oxygen species
SD-OCT: Spectral Domain Optical Coherence Tomography
SRC: Spare respiratory capacity
TCA: Tricarboxylic acid
