# Supplementary material for: Glucose transporter 1 critically controls microglial activation through facilitating glycolysis
Source: Mol Neurodegener. 2019 Jan 11;14:2. doi: 10.1186/s13024-019-0305-9 (PMC6329071; doi:10.1186/s13024-019-0305-9)
Supplement: Supplementary file 1 — Figure S1. ATP-induced microglial migration using the transwell assay. B6M7, BV2 cells and peritoneal macrophages (3,000/well) were seeded into the upper chamber in a trans-well culture system for 16h. 100 μM ATP was then added into the bottom chamber. 3 hours later, cell numbers in bottom chambers were counted. Mean ±SEM, N=6 ***P<0.001. Figure S2. The effect of STF31 on microglial cells. (A) B6M7 cells were treated with different concentrations of STF31 and cell viability was assessed by AlamarBlue 24 h and 48 h after treatment. N=3. (B) Representative images and quantifications of TUNEL assay in naïve, M(LPS+IFNγ), and M(IL-4) microglia with or without 5 μM STF31. Arrow shows a TUNEL-positive cell. Scale bar = 50 μm. N=3. (C, D) RT-PCR (C) and Western Blot (D) showing the expression of GLUT1 in B6M7, with or without 5 μM STF31. (E) RT-PCR showing the expression of GLUTs in B6M7 microglial cells, with and without 5 μM STF31. RT-PCR and western blot data were expressed as relative expression against β-actin (n=3). Mean ±SEM. Figure S3. The effect of STF31 on mouse retina. Mice were injected intraperitoneally with STF31 (10 mg/kg) for 5 days. Four mice were used. (A) Representative ERG responses at day 0 (before the onset of STF31 treatment) and day 4. (B) Quantitative analysis of a-wave and b-wave amplitudes, before (day 0) and after (day 4) STF31 treatment. (C, D) Representative images (C) and quantifications (D) of TUNEL assay in control and STF31-treated mice. Scale bar = 25 μm. Figure S4. The effect of STF31 on retinal thickness in light-induced retinal degeneration. CX3CR1gfp/+ mice were exposed to 50,000 lux focal white light for 10 minutes with daily STF31 or vehicle (DMSO) treatment. (A-D) Representative images from each group of mice. (E) Bar graph showing the average thickness of the entire neuronal retina (from nerve fiber/ganglion layer to inner/outer segments layer, illustrated in A) in each group. (F) Bar graph showing the thickness of the inner retin [file 13024_2019_305_MOESM1_ESM.docx]

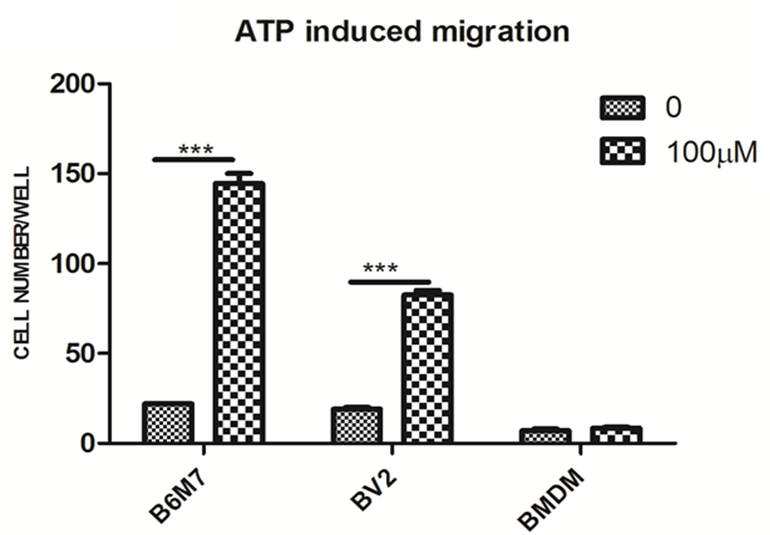


#### **Figure S1. ATP-induced microglial migration using the transwell assay.**

B6M7, BV2 cells and peritoneal macrophages (3,000/well) were seeded into the upper chamber in a trans-well culture system for 16h. 100 μM ATP was then added into the bottom chamber. 3 hours later, cell numbers in bottom chambers were counted. Mean ±SEM, N=6 ***P<0.001.


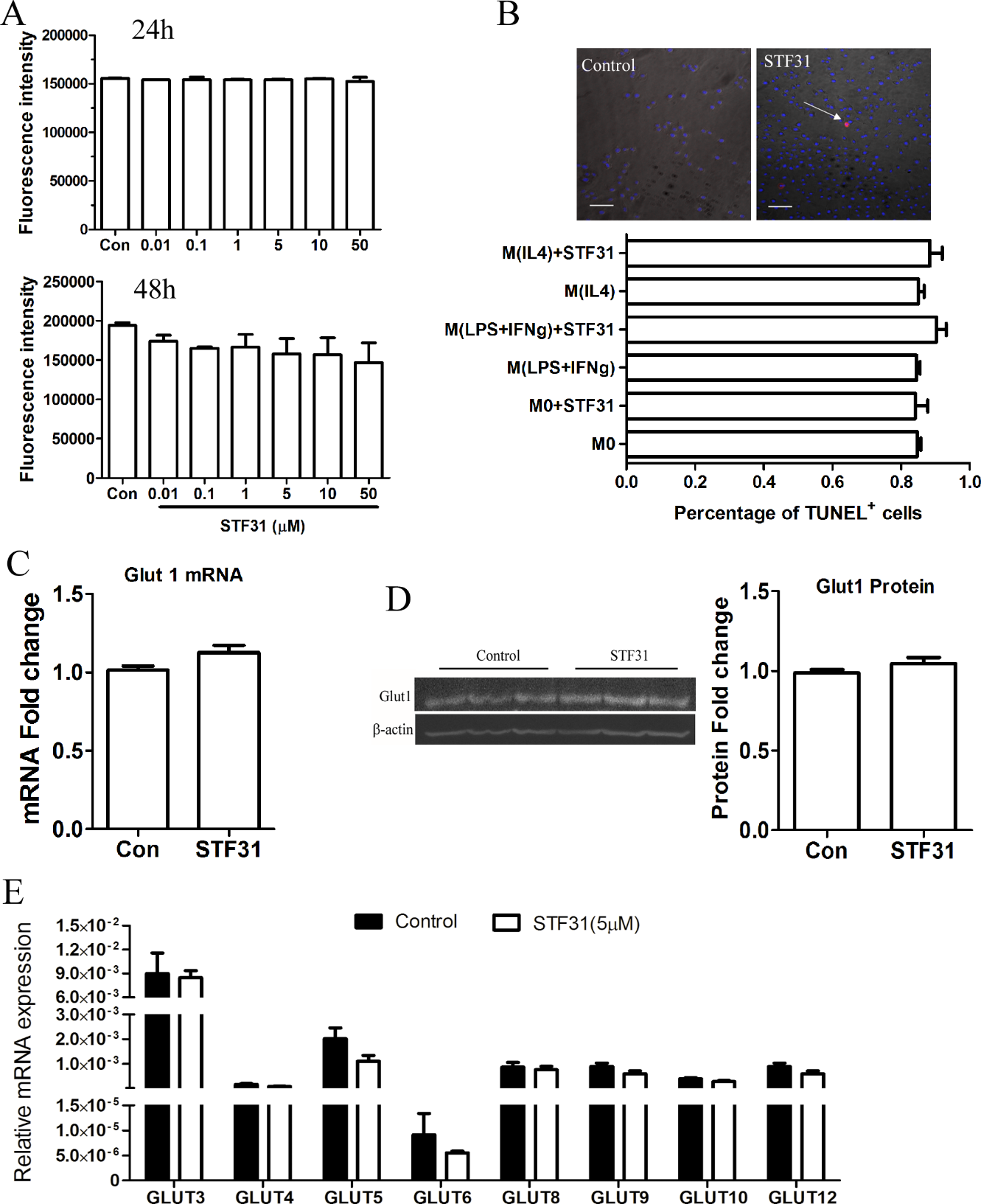


**Figure S2. The effect of STF31 on microglial cells.** (A) B6M7 cells were treated with different concentrations of STF31 and cell viability was assessed by AlamarBlue 24 h and 48 h after treatment. N=3. (B) Representative images and quantifications of TUNEL assay in naïve, M(LPS+IFNγ), and M(IL4) microglia with or without 5 μM STF31. Arrow shows a TUNEL-positive cell. Scale bar = 50 μm. N=3. (C, D) RT-PCR (C) and Western Blot (D) showing the expression of GLUT1 in B6M7, with or without 5 μM STF31. (E) RT-PCR showing the expression of GLUTs in B6M7 microglial cells, with and without 5 μM STF31. RT-PCR and western blot data were expressed as relative expression against β-actin (n=3). Mean ±SEM.


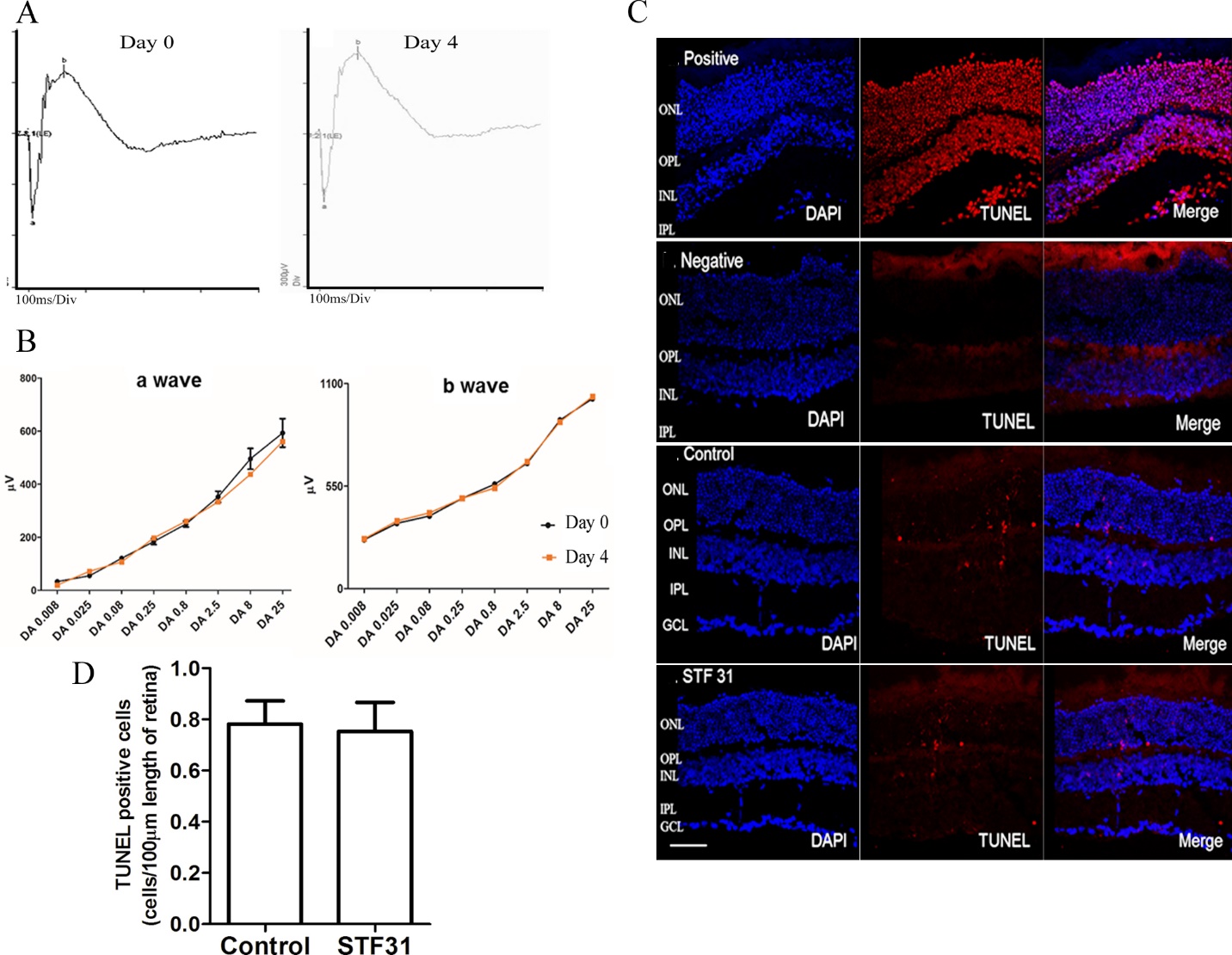


**Figure S3. The effect of STF31 on mouse retina.** Mice were injected intraperitoneally with STF31 (10 mg/kg) for 5 days. Four mice were used. (A) Representative ERG responses at day 0 (before the onset of STF31 treatment) and day 4. (B) Quantitative analysis of a-wave and b-wave amplitudes, before (day 0) and after (day 4) STF31 treatment. (C, D) Representative images (C) and quantifications (D) of TUNEL assay in control and STF31-treated mice. Scale bar = 25 μm.


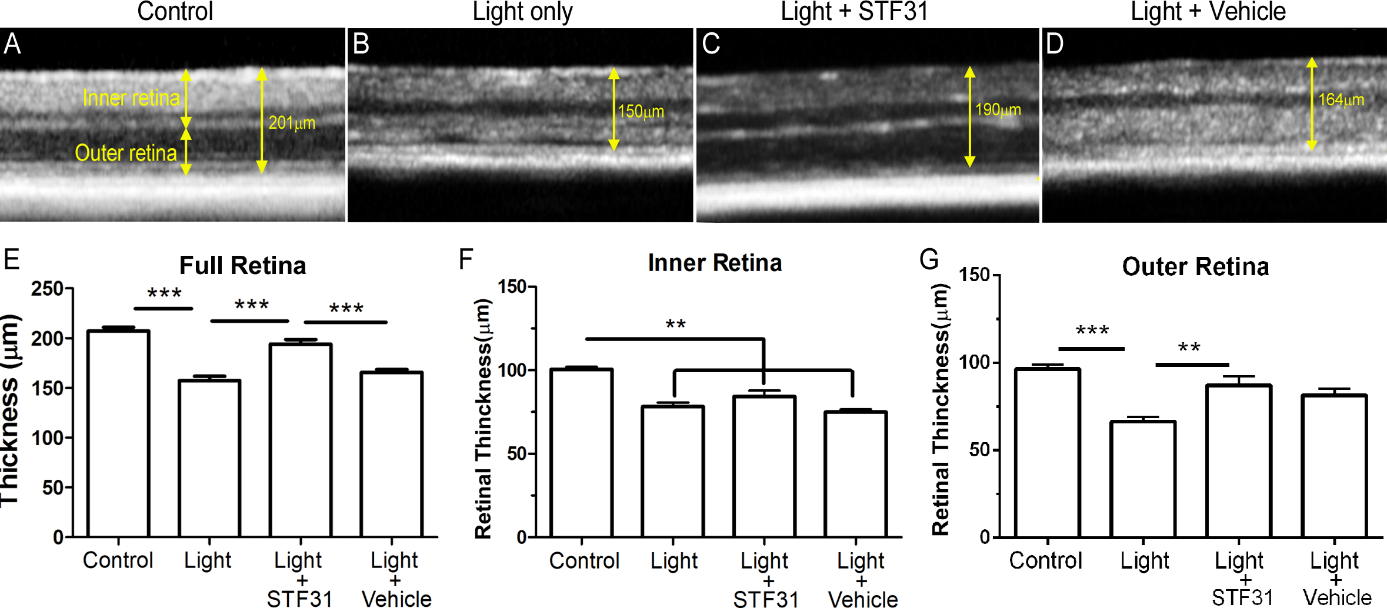


**Figure S4. The effect of STF31 on retinal thickness in light-induced retinal degeneration.** CX3CR1^gfp/+^ mice were exposed to 50,000 lux focal white light for 10 minutes with daily STF31 or vehicle (DMSO) treatment. (A-D) Representative images from each group of mice. (E) Bar graph showing the average thickness of the entire neuronal retina (from nerve fiber/ganglion layer to inner/outer segments layer, illustrated in A) in each group. (F) Bar graph showing the thickness of the inner retina (from nerve fiber/ganglion layer to inner plexiform layer, illustrated in A). (G) Bar graph showing the thickness of the outer retina (from outer nuclear to inner/outer segments, illustrated in A). Full control: N=4 mice, N=6 mice in other groups. **P<0.01; ***p < 0.001.
